# Integrative Computational Chemistry Approaches in Modern Drug Discovery: Advances in Docking, Pharmacophore Modeling, Molecular Dynamics, and Virtual Screening

**DOI:** 10.3390/pharmaceutics18050565

**Published:** 2026-05-01

**Authors:** Ali Altharawi, Safar M. Alqahtani

**Affiliations:** Department of Pharmaceutical Chemistry, College of Pharmacy, Prince Sattam Bin Abdulaziz University, Al-Kharj 11942, Saudi Arabia; safar.alqahtani@psau.edu.sa

**Keywords:** molecular docking, pharmacophore modeling, molecular dynamics, virtual screening, free-energy calculations, structure prediction

## Abstract

Computational chemistry has played a central role in early-stage drug discovery by accelerating target selection, hit identification, and lead optimization. This review summarizes recent developments in molecular docking, pharmacophore modeling, molecular dynamics (MD), and virtual screening (VS), with a focus on their application in practical drug discovery workflows. Advances in docking protocols, including consensus scoring, physics-based rescoring, and ensemble approaches, addressed the challenges of receptor flexibility. Both ligand-based and structure-based pharmacophore models facilitated scaffold hopping and guided library prioritization. MD simulations were used to assess binding pose stability, identify cryptic binding pockets, and characterize solvent interactions. These simulations also supported free-energy calculations using endpoint and alchemical methods. Large-scale VS campaigns employed curated compound libraries, often composed of make-on-demand molecules, and relied on high-performance computing or cloud infrastructure to screen up to 10^9^ compounds. Hits were validated using orthogonal biophysical assays and filtered by absorption, distribution, metabolism, excretion, and toxicity (ADMET) predictions. Integrated pipelines combining pharmacophore modeling, docking, MD, and free-energy calculations improved enrichment rates and reduced the number of compounds requiring synthesis. Several case studies demonstrated the identification of nanomolar-affinity leads from ultra-large screening campaigns. The review also addressed ongoing challenges, such as inconsistent scoring of binding affinity, protonation, and tautomeric errors, dataset bias, and reproducibility issues. Strategies to mitigate these limitations included standardized library preparation, adherence to FAIR (Findable, Accessible, Interoperable, and Reusable) data principles, and the use of prospective benchmarking protocols. The review discussed emerging trends, including the use of quantum chemistry for electronic structure refinement, ensemble docking guided by cryo-electron microscopy (cryo-EM) data, and the integration of computational tools with automated synthesis and high-throughput screening in closed-loop discovery systems. These approaches have the potential to accelerate the design–make–test cycle, increase hit novelty, and improve decision-making in early drug development programs.

## 1. Introduction

The process of drug discovery is complex and resource-intensive, historically involving both empirical screening approaches and rational drug design strategies, including early medicinal chemistry and structure-guided optimization [1]. Drug discovery remains one of the most time-intensive, expensive, and high-risk processes in biomedical research, and, on average, total costs exceed USD 2.6 billion, with timelines averaging 10–15 years per compound that receives market approval [2]. Despite significant investments in drug discovery, the attrition of drug candidates remains incredibly high, and reportedly only 1 in every 5000–10,000 compounds receives approval for market use [3].

In this challenging landscape, computational chemistry has become an integral component of the drug discovery pipeline, offering cost-effective, rapid alternatives to early-stage experimental screening [4,5]. Drug discovery starts with target identification and proceeds sequentially through lead discovery, optimization, preclinical, and clinical trials. At least through the first three stages of drug discovery, computational tools play an important role by helping prioritize hits and optimize molecular interactions [6]. Computational chemistry enables virtual screening of millions of compounds, simulation of protein–ligand dynamics, and prediction of absorption, distribution, metabolism, excretion, and toxicity (ADMET) profiles [7]. As experimental screening campaigns often involve high-throughput systems with limited throughput, in silico methods significantly reduce attrition by removing weak candidates before costly biological testing [8].

Structure-based drug design (SBDD) took root in the 1980s following the emergence of crystallographic techniques, but the computational revolution of the last two decades has made molecular modeling accessible to a much wider range of applications [9]. With the exponential increase in structural and chemical databases and the integration of artificial intelligence, in silico platforms are now capable of screening billions of compounds in a matter of days [4,10]. The relevance of these computational methods has increased further during pandemics and emerging disease outbreaks, where time-to-discovery is critical [2]. Recent advances such as AlphaFold 3 significantly extend structure prediction capabilities beyond single proteins, enabling accurate modeling of protein–ligand, protein–nucleic acid complexes, and post-translational modifications. These developments enhance the applicability of structure-based drug design by providing more realistic interaction models [11,12]. Modern approaches in the structure prediction, especially the development of AlphaFold 3 and diffusion-based predictors, have made the field of computational drug discovery much broader and are not limited to the performance of single-protein systems. The goals of these methods are to simulate more and more complicated biological assemblies, such as protein–ligand interactions, protein–nucleic acid complexes, and systems with post-translational modifications. These methods combine principles of deep learning with those of structural biology, which provides the possibility of bridging the gaps between the prediction of static structures and the dynamics of molecular recognition processes, which underlie the structure-based design of drugs in targets that were previously challenging. Nevertheless, even with these encouraging trends, generalizable and precise modeling of such complex systems is still a research area. Recent methods, such as AlphaFold 3, have proven to make significant strides, but nonetheless, are limited in their ability to predict binding affinities, ligand conformations, and interaction energetics in a variety of chemical and biological systems with low confidence. Specifically, issues with the capture of induced-fit effects, solvent interactions, and the effect of post-translational modifications on binding interfaces remain. Likewise, diffusion-based generative models, though strong in investigating structural and chemical space, need additional validation to guarantee their robustness and reproducibility in future applications. In turn, these new approaches are to be considered as complementary tools, not as substitutes for the old computational and experimental approaches. The combination of structure prediction models with the use of molecular docking, molecular dynamics simulations, and experimental validation is necessary to enhance reliability and interpretability. Further methodological development, comparison with high-quality datasets, and future validation experiments will be essential in order to maximize the potential of these methods in drug discovery pipelines. Among the most prominent computational approaches, molecular docking simulates the binding affinity between ligands and biological targets and is a foundational tool in virtual screening workflows [13]. Several docking algorithms, including AutoDock, GOLD, and Glide, have been fine-tuned to enhance overall binding mode prediction and scoring functions [14]. Pharmacophore modeling, another pivotal methodology, allows abstraction of critical features necessary for molecular recognition and is frequently utilized in the absence or limitations of crystal structures [15]. Both structure-based pharmacophores and ligand-based pharmacophores can also be incorporated into virtual screens to enhance the overall accuracy and minimize false positives. Molecular dynamics (MD) simulations provide atomistic perspectives of protein–ligand complexes over time, revealing conformational changes and confirming docking-predicted poses [8]. Rapid MD methods like accelerated MD and meta dynamics, along with the advantages of GPU-based parallelization, have made these simulations not only more accessible but also more accurate [16]. The integration of MD simulations, pharmacophore models, and docking provides a more realistic framework for studying drug–target interactions [17]. Virtual screening has become the procedure for filtering ultra-large libraries, enabling ligand- and structure-based workflows to be performed with high throughput and accuracy to rank or prioritize compounds for synthesis or testing [18]. Recent innovations in AI-guided screening, deep generative models, and transfer learning have led to adaptive systems that can design new compounds from scratch (e.g., de novo) [19]. Combining several computational methods has led to more robust workflows, with high hit-to-lead conversion rates and low attrition rates at late stages observed [19]. In light of these developments, computational methods have become indispensable tools not just for speeding drug discovery but also for improving the precision, safety, and affordability of drugs [20]. However, there are still limitations on accuracy, reliability, and generalizability that hinder universal acceptance [21]. This review examined recent advances in established computational chemistry methods, including molecular docking, pharmacophore modeling, molecular dynamics, and virtual screening, and their uses and limitations in practice for drug discovery pipelines. Another objective was to provide an overview of the connectivity between computational approaches and how integrating several methods can speed up the drug discovery process, offering powerful tools to address longstanding challenges in lead identification and optimization, thus setting the stage for the detailed discussions that follow in this review.

## 2. Computational Approaches in Drug Discovery

Computational chemistry has emerged as an essential pillar of modern drug discovery, significantly accelerating design–make–test–analyze cycles and reducing experimental effort across target identification, hit finding, and lead optimization. Structure-based methods, such as molecular docking and molecular dynamics (MD) simulations, are now increasingly intertwined with ligand-based approaches, such as pharmacophore modeling and cheminformatics-guided virtual screening (VS). Mature public data repositories and advances in structure prediction have vastly expanded the feasible problem space from thousands to billions of compounds and from single-protein to pathway- and network-level predictions. These advances enable tighter integration between in silico hypotheses and orthogonal biophysical and cellular assays, improving the precision and reproducibility of medicinal chemistry campaigns [22,23].

Structure-based screening typically begins with a high-quality receptor model derived from crystallography, cryo-EM, or NMR deposited in the Protein Data Bank (PDB). Docking engines (for example, AutoDock Vina and Glide) rapidly generate and score poses for millions of ligands, providing prioritized hit lists for purchase or synthesis [24,25]. In practice, computational triage is directly coupled to experimental validation through using biophysical assays such as differential scanning fluorimetry (DSF), surface plasmon resonance (SPR), and isothermal titration calorimetry (ITC) and cell-based readouts; iterative feedback refines receptor protonation states, binding-site water treatment, and ligand tautomers before escalating promising scaffolds into prospective structure-activity relationship studies [26]. MD simulations then probe pose stability, desolvation, and protein flexibility on nanosecond–microsecond timescales, highlight cryptic pockets, and support free-energy calculations to rationalize affinity changes observed in the bench campaigns [23]. Ligand-based methods complement this loop when structural data are sparse or when chemotypes are diversified around known actives. Pharmacophore modeling distills essential 3D features (e.g., H-bond donors/acceptors, hydrophobes, aromatic centroids) required for activity, enabling scaffold hopping and fast database queries (Table 1); platforms like PHASE couple pharmacophore perception with 3D QSAR to guide substituent changes with quantitative predictions [27]. To control attrition due to poor ADME, rule-based filters, most prominently Lipinski’s “Rule of Five”, flag liabilities early and focus synthesis on developable regions of chemical space without over-constraining novelty [28].

**Figure 1:**
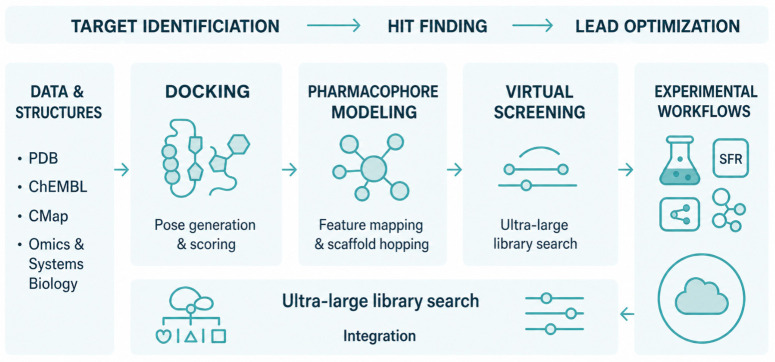
Workflow from target identification to lead optimization. Data resources feed docking, pharmacophore modeling, and virtual screening. Integration and experimental workflows provide feedback, enabling ultra-large library search and prioritization of chemotypes.

At the target-identification stage, transcriptomic perturbation resources such as the Connectivity Map (CMap/LINCS) allow researchers to link compounds, genes, and diseases by shared expression signatures (Figure 1). These signatures can uncover actionable mechanisms and prioritize targets for phenotypic hits when genetics or pathway evidence is equivocal. Once targets are selected, structure-enabled campaigns exploit high-resolution complexes and, increasingly, AI-predicted structures to expand coverage beyond traditionally tractable protein families. Docking and shape/pharmacophore screens seed initial hits; consensus scoring and rescoring with physics-based or ML-augmented models improve enrichment. Hit-to-lead optimization then leverages MD for water-network analysis, conformational selection, and binding-mode stabilization, informing substituent choices that balance potency and physicochemical properties. Throughout, medicinal chemistry teams integrate developability heuristics (e.g., RO5 compliance and its known exceptions) with iterative synthesis to avoid late-stage failures [23,28]. Large-scale virtual screening has matured from millions to billions of candidates via cloud and HPC platforms, workflow automation, and fault-tolerant orchestration. VirtualFlow exemplifies this shift, enabling ultra-large docking campaigns with modular support for multiple docking engines and robust pre-processing, which in turn has led to prospective validations against diverse targets. Open bioactivity repositories such as ChEMBL supply target-annotated structure–activity data that seed pharmacophore/ligand-based models, enable decoy generation, and support external validation of computational pipelines [40]. Early computer-aided design leaned heavily on interpretable rules and linear models, with RO5 serving as a pragmatic filter for oral drug-likeness during lead optimization [28]. Docking programs evolved in parallel, improving sampling and scoring through knowledge-based potentials and empirical terms; method benchmarks on Vina and Glide demonstrated substantial gains in pose prediction and enrichment versus earlier tools [22]. Over the last decade, two inflection points reshaped the field. First, routine microsecond-scale MD powered by algorithmic and hardware advances made it practical to account for receptor dynamics, allostery, and water rearrangements in design decisions rather than treating the protein as static [23]. Second, AI-based structure prediction achieved near-experimental accuracy for many single-chain proteins, vastly expanding the structural coverage for previously “undockable” targets and enabling downstream SBDD at scale [41]. These methodological shifts catalyzed new workflows: predicted structures can be refined by MD, cross-checked against homology models, and used directly for grid generation and docking; ambiguous binding-site residues can be enumerated across protonation/tautomer states and validated by pose stability. Pharmacophore hypotheses extracted from known ligands can be projected onto AI-predicted pockets to rationalize activity cliffs. In parallel, ML models trained on curated ChEMBL bioactivity and assay metadata guide active-learning loops, prioritize syntheses, and calibrate uncertainty, while expression-signature matching from CMap/LINCS links chemical perturbations to pathway rewiring for mechanism-of-action inference and indication expansion [40]. Progress in computational discovery is inseparable from the growth of high-quality public datasets and shared infrastructure. The PDB standardizes macromolecular structures, enabling consistent binding-site preparation, water and ion curation, and comparative modeling pipelines. ChEMBL delivers assay-level activity annotations crucial for kinetic vs. equilibrium distinctions, target engagement confidence, and negative data ingredients for reproducible model training and unbiased external validation. Expression-profiling resources such as the modern CMap make it feasible to connect small molecules to genetic programs, triage series by pathway selectivity, and anticipate liabilities by monitoring off-target transcriptional fingerprints [24,25,40].

## 3. Molecular Docking: Theory, Tools, and Applications

Molecular docking aims to predict how a ligand binds within a macromolecular target and to estimate the strength of this interaction. Typical workflows comprise (i) receptor and ligand preparation (protonation/tautomer assignment, conformer generation, binding-site definition), (ii) pose generation via systematic, stochastic, or knowledge-guided search, and (iii) scoring and ranking of poses using physics-based, empirical, or knowledge-based functions. Most docking programs treat the receptor as rigid or semi-flexible (sidechain rotamers or soft potentials), while the ligand explores translation, rotation, and torsions; induced fit is approximated by sidechain sampling or post-docking relaxation. Scoring functions balance terms for van der Waals complementarity, electrostatics, desolvation, and sometimes hydrogen bonding and metal coordination; consensus or rescoring strategies are often used to mitigate function-specific bias. Docking paradigms divide broadly into protein–ligand docking (typical drug-like molecules) and protein–protein docking (PPDock), the latter contending with far larger interfaces, rugged energy landscapes, and pronounced conformational change. For PPDock, sampling schemes must address multiscale translations and rotations of both partners, often guided by experimental restraints (e.g., XL-MS, NMR, cryo-EM) [22,42]. Multiple mature platforms underline the docking ecosystem. For instance, AutoDock/AutoDock Vina implemented an efficient stochastic search with multithreading and a reparametrized empirical scoring function that has served as one of the backbones of structure-based virtual screening (SBVS) and pose prediction. Glide combined hierarchical filtering with exhaustive pose refinement, as well as proprietary scoring (GlideScore), including strong early enrichment in SBVS benchmarks [22]. GOLD used a genetic algorithm to improve pose accuracy in challenging pockets by combining the flexibility of the protein’s side chains with user-specified constraints. Earlier releases of DOCK introduced a grid-based shape-complementarity approach and anchor-and-grow strategies, which eventually evolved into a modular suite widely used in academia [29]. For PPDock, RosettaDock enabled rigid-body sampling and optimizing some side-chain degrees of freedom within a Rosetta energy function, and it is accessible through an automated web server [42]. This suite of available dockers ranges from free/open-source (AutoDock Vina, DOCK) to commercially available tools (Glide, GOLD) and from command line engines to user-friendly servers for workflows ranging from exploratory screens of small sizes to ultra-large screen campaigns [22,29,42]. Ensemble docking addresses receptor plasticity by docking against multiple conformations derived from experimental structures, homology models, or molecular dynamics (MD) trajectories and aggregating results via consensus scoring or clustering. This strategy improves the chance of sampling near-native poses in flexible or allosteric sites and has shown practical benefits across GPCRs, kinases, and viral enzymes [43]. Deep learning for pose prediction and scoring is increasingly integrated into docking. Convolutional neural networks trained on protein–ligand grids can rescore poses to improve top-ranked accuracy without changing the search engine; GNINA 1.0, for example, couples CNN scoring to Vina-like sampling and demonstrates consistent pose improvements across standard benchmarks [44]. Beyond CNN rescoring, data-efficient graph and 3D-grid models continue to expand coverage to new protein families. QM/MM integration augments empirical scoring by recalculating key poses with quantum mechanical (QM) methods, capturing polarization, charge transfer, metal coordination, and covalent mechanisms that classical force fields struggle to model. Used selectively (e.g., rescoring top poses or refining ambiguous chemotypes), QM or QM/MM can correct rankings and reduce false positives, albeit at a higher cost [45]. These advances close the distance between rate and physical realism and lend a greater degree of reliable prospective design when combined with orthogonal filters (e.g., pharmacophore constraints, a water thermodynamics model, or an ADME predictor based on machine learning [43,44,45].

Recent advancements in docking include tools such as SMINA, QuickVina, DiffDock, and GNINA, which incorporate improved scoring functions and machine learning-based pose prediction. Diffusion-based docking methods, such as DiffDock, further enhance pose accuracy by modeling ligand placement as a generative process [46,47].

**Figure 2:**
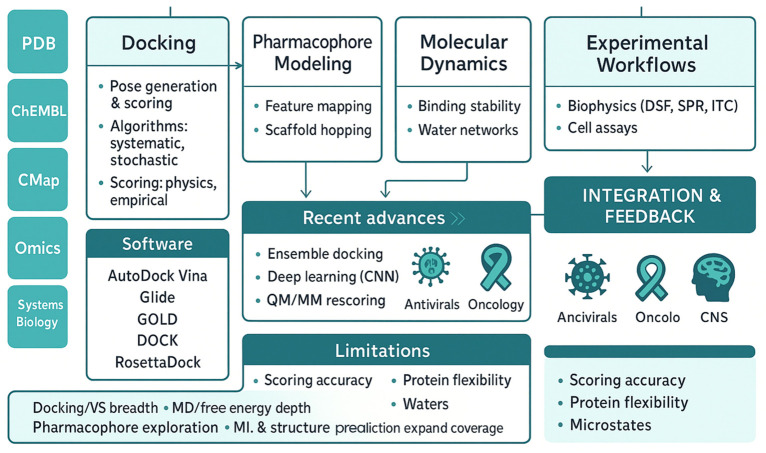
Framework linking data repositories to docking, pharmacophore modeling, and molecular dynamics, supported by experimental workflows. Panels summarize software, advances, limitations, with integration feedback highlighting antiviral, oncology, and CNS key applications.

Docking permeates multiple phases of drug discovery. In the field of antivirals, docking was instrumental in triaging and prioritizing inhibitors of the SARS-CoV-2 main protease (Mpro) following crystal structure availability, highlighting SBVS in the hit identification phase and then medicinal chemistry optimization phase; this was also the first or most extensive report of working within the framework of SBVS to combine structure determination and discovery of inhibitors, further detailing structure–activity relationships and demonstrating how SBVS would further support atomically precise approaches during hit discovery [48]. In the CNS area, docking to GPCRs provides novel chemotypes; structure-based docking to the μ-opioid receptor assisted the discovery of PZM21, a Gi-biased agonist that provokes less respiratory depression in preclinical models (Figure 2), demonstrating how docking can leverage considerations for scaffolds with specific signaling profiles [49]. In oncology, docking campaigns commonly seed a series of kinase inhibitors and engage target protein–protein interactions (e.g., BCL-2 family, KRAS effectors), iteratively ranking members of fragments inhibited undefined hot spots, frequently followed by an induced-fit or MD refinement to assess selectivity. Successful efforts across therapeutic areas have been associated with careful target preparation (e.g., suitable biologically relevant protonation states of the molecule, metal or halogen treatment), diligent library design (physicochemical and substructure filters), and careful post-docking adjudication (pharmacophore fit, strain energy, water placement, QM/MM rescoring, experimental orthogonality, etc.) [48,49]. Scoring inaccuracies remain the primary bottleneck: empirical functions trade speed for approximations to solvation and entropy, leading to false positives/negatives and limited rank-order correlation with affinity. Protein flexibility and waters are imperfectly captured by single-structure docking; key rearrangements, cryptic pockets, or displaceable waters can defeat rigid-receptor assumptions. Chemistry edge cases, including covalent mechanisms, transition metals, tautomers/protonation microstates, halogen bonding, and pi-stacking anisotropy, require special handling. Dataset bias and reproducibility also matter. Retrospective enrichments may not translate prospectively if benchmarks overlap with training data (for ML-augmented scorers) or if preparation pipelines differ. Looking forward, several directions are promising. First, physics-aware ML hybrid models that preserve geometric and energetic constraints while learning from large structural corpora should improve pose plausibility and generalization beyond familiar pockets. Second, ensembles and water thermodynamics (e.g., GCMC water, water networks) will likely become standard in SBVS triage. Third, selective QM/MM rescoring and polarizable force fields can correct borderline decisions, especially for metalloproteins and covalent inhibitors. Workflow integration from pocket detection to docking to FEP/MD refinement, plus better uncertainty quantification and prospective benchmarking, will continue to raise confidence in docking-driven nominations [43,44,45].

## 4. Pharmacophore Modeling: From Concept to Clinical Candidates

**Figure 3:**
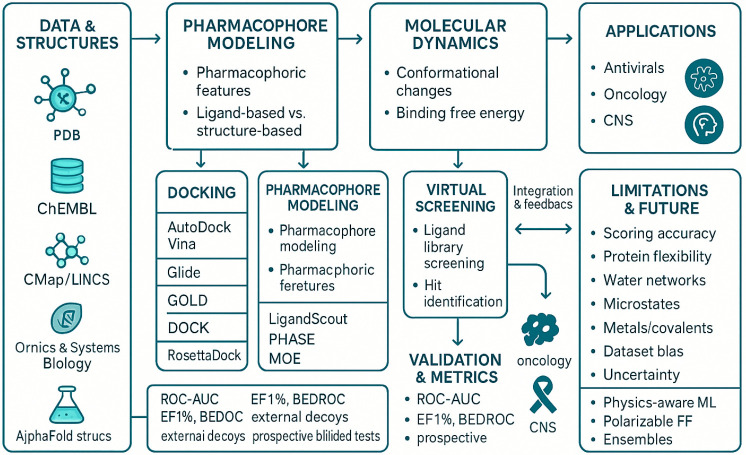
Comprehensive schematic linking data sources to pharmacophore modeling, molecular dynamics, virtual screening, and docking, plus validation metrics. Arrows depict integration feedback, with applications and limitations: scoring, flexibility, waters, metals, uncertainty.

A pharmacophore is the ensemble of steric and electronic features that a ligand must present to ensure optimal interactions with a biological target and elicit a response; common features include hydrogen-bond donors/acceptors, hydrophobes, aromatics, cations/anions, and metal binders [50]. In ligand-based modeling, multiple known actives are aligned to derive a consensus feature pattern that tolerates scaffold diversity and supports scaffold hopping. In structure-based modeling, features are extracted from a receptor–ligand complex (or a prepared apo pocket), often complemented by water and protonation analysis to refine the geometry and priorities of interaction hotspots (Figure 3). In practice, teams use ligand-based hypotheses for rapid library triage and structure-based (SB) models to rationalize SAR and steer substituent placement near obligatory interactions or displaceable waters, with the two approaches frequently combined in iterative cycles. Several mature tools support both hypothesis generation and large-scale screening. LigandScout derives 3D pharmacophores directly from protein-bound ligands and exports queries for fast shape/feature search. PHASE integrates common-pharmacophore identification, hypothesis scoring, and 3D-QSAR, and is widely used to couple pharmacophores with machine-learned activity models [27].

MOE provides comprehensive conformer generation and pocket/feature utilities; methods such as LowModeMD improve conformational sampling for alignment and hypothesis robustness [31]. For rapid, free ligand-based hypothesis building, PharmaGist aligns multiple ligands to identify common feature constellations. These platforms underpin both quick exploratory filters and production-grade virtual screening pipelines. Because pharmacophore hits are typically prioritized from very large libraries, early-recognition metrics are essential. Receiver-operating characteristic (ROC-AUC) provides a global view, but enrichment-focused measures such as EF at low database fractions and BEDROC more faithfully evaluate real-world screening, where only the top fraction is tested. Robust practice includes external decoy sets, retrospective recovery of known actives, and prospectively blinded tests that fix decision thresholds before synthesis.

**Figure 4:**
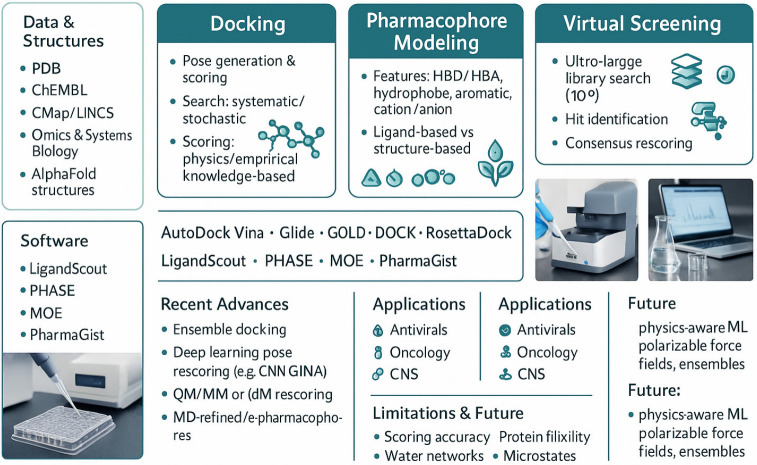
Data sources, docking, pharmacophore modeling, and virtual screening with representative software. Lower panels present advances, applications, and limitations; laboratory photographs illustrate assay context alongside computational outputs.

Energy-optimized or e-pharmacophores project per-residue or per-feature energetic terms from a receptor–ligand complex onto pharmacophoric features, producing hypotheses that better reflect the underlying physics than purely geometric models. They are often used to re-rank or prune docking hits before. MD-refined pharmacophores incorporate receptor dynamics by extracting features from conformational ensembles; compared with single-snapshot models, MD-derived hypotheses can better separate actives from decoys and reveal transient/cryptic interactions. Beyond physics-based refinements, AI-enhanced pharmacophore workflows now condition generative or predictive models on 3D feature maps, improving control during linker/R-group design and accelerating hypothesis testing [51]. These strategies increase hit quality by reconciling speed (feature search) with realism (energetics and dynamics). Pharmacophore queries routinely seed phenotypic-to-target campaigns (back-mapping features from chemotyped hits), guide scaffold hopping with preserved interactions in GPCRs/kinases, and pre-filter ultra-large libraries before docking. They are especially valuable when target structures are incomplete or flexible, and when medicinal chemistry requires diverse chemotypes with shared binding logic (Figure 4). Key limitations persist. The hypotheses may overfit sparse training sets; feature geometry can be sensitive to conformer quality; and static models can miss water-mediated or induced-fit interactions unless augmented by MD or energetic analysis. Best practice combines ligand- and structure-based evidence, validates with enrichment-focused metrics (Table 2), and closes the loop with prospective assays to refine features and tolerances.

## 5. Molecular Dynamics (MD) Simulations: Capturing Flexibility and Dynamics

Molecular dynamics (MD) provides time-resolved, atomistic movies of biomolecules by integrating Newton’s equations of motion on a potential energy surface defined by a force field. For protein and protein–ligand complex, modern biomolecule-tuned force fields CHARMM36m, AMBER ff14SB, and OPLS3e deliver improved secondary-structure balance, sidechain rotamer distributions, and small-molecule compatibility, enabling routine simulations from nanoseconds to multi-microseconds on commodity GPUs [23,33,34,35]. MD complements static models by revealing conformational selection, induced fit, cryptic pocket formation, and ordered water networks that strongly modulate ligand binding and selectivity [23].

Conventional MD uses a fixed potential and timestep to sample thermally accessible motions and is the backbone for stability assessments of docked complexes. Enhanced sampling methods accelerate rare events. Accelerated MD lowers effective energy barriers to promote transitions between metastable states, improving exploration of loops, side chains, and allosteric sites [60]. Steered molecular dynamics (MD) utilizes time-dependent forces to study unbinding trajectories, rupture forces, or conformational switching. These studies typically yield mechanistic hypotheses that are addressed through mutagenesis or linker design. Meanwhile, in thermodynamic studies, end-point free energy approaches with MD files, such as MM-PBSA or MM-GBSA, can provide estimates of relative binding affinity and serve as efficient triage methods when coupled with adequate dielectric and entropy approximations [61]. Alchemical free energy methods (FEP) can be even more accurate in determining affinity, as they more accurately convert ligands along non-physical paths. Furthermore, more recently, when the workflow provides sufficiently rigorous sampling and error control, FEP can provide future estimates with sub-kilo-caloric precision for congeneric series of ligands. While many academic and commercial docking software can generate candidate poses, MD directly assesses the physical plausibility of these poses and resolves ambiguities arising from protonation, tautomerism, or the placement of waters between the ligand and the target. Additionally, while simulations may run for long periods, settings as short as 100 ns can be used to develop hypotheses for molecular design; in fact, shorter simulations may obviate strained poses, quantify hydrogen bond persistence, or reveal water-mediated networks. Clustering of MD frames can yield enriched pharmacophore hypotheses that account for or quantify dynamic donor/acceptor and hydrophobic patterns not accessible from a single snapshot [23]. For prioritization, end-point methods (MM-GBSA/MM-PBSA) or targeted FEP runs on MD-relaxed poses help rank series and rationalize structure–activity relationship, while steered MD provides qualitative rankings for unbinding kinetics in transporters and GPCRs [61]. Machine learning is increasingly used to compress, classify, and predict behavior from large trajectories. Markov state models (MSMs) coarse-grain dynamics into metastable states connected by transition probabilities, enabling estimates of rates, pathways, and long-timescale observables from many short simulations [62]. Deep-learning architectures such as VAMPnets learn slow collective variables and state decompositions directly from coordinates, improving the objectivity and reproducibility of clustering and facilitating mechanism discovery and rare-event prediction [63]. These methodologies may identify an event representing a pocket opening, find ligand able conformations for ensemble docking, or even pinpoint conformers appropriate for free-energy calculations focused on a particular pathway, enabling more accurate selection of conformations for downstream free-energy calculations. The unique advantages of MD are its physical interpretability and its temporal resolution. The physical interpretability of MD can help answer questions about why a pose has good docking stability, how water reorganizes, and which conformations an allosteric modulator stabilizes. The challenge with any predictive power lies in the accuracy of the force field, sampling techniques, and system preparation. Some common artifacts of MD may be due to imprecise salt bridges, misprotonation of residues or ligands, unstable ligation of metals or analytes, or an otherwise insufficient ion or lipid composition. Practical strategies include (i) selecting a force field validated for the system class (CHARMM36m, AMBER ff14SB, OPLS3e), (ii) building ensembles of starting structures (multiple receptor conformations and ligand tautomers), (iii) monitoring stability with RMSD/RMSF, hydrogen-bond occupancy, and water residence times, (iv) using enhanced sampling when functional transitions are slow (accelerated or steered MD), and (v) reserving rigorous free-energy methods for late-stage prioritization where small potency differences matter [33,34,35,60,61]. With these safeguards and ML-assisted analysis, MD now routinely elevates docking and pharmacophore efforts from pose generation to mechanism-aware design [23,62,63].

## 6. Case Studies of Computational Drug Discovery

Several successful drugs have been developed using computational approaches. Representative examples are summarized below in Table 3.

## 7. Virtual Screening: Large-Scale Compound Prioritization

Virtual screening (VS) prioritizes candidates from massive chemical libraries by computationally estimating their likelihood to bind a biological target. In practice, VS takes two complementary forms. Ligand-based VS (LBVS) infers activity from molecular similarity, pharmacophoric patterns, or learned embeddings of molecules with known activity; it excels when target structures are uncertain, but the Structure Activity Relationship is rich. Structure-based VS (SBVS) relies on protein structures to dock and score candidates in silico; it is most informative when high-quality experimental or predicted (e.g., AlphaFold) structures are available. Modern discovery programs commonly use both LBVS to rapidly down-select ultra-large libraries and SBVS (docking, rescoring, pose filtering) to refine the final shortlist. Public and commercial repositories now routinely supply billions of purchasable or make-on-demand molecules (e.g., ZINC20/22, PubChem, ChEMBL, and enumerated universes such as GDB-17), enabling VS at unprecedented scale (Table 4). A typical SBVS pipeline proceeds as: (1) library acquisition/design and curation; (2) physicochemical and liability filtering (e.g., Lipinski/Veber-style criteria, medicinal chemistry rules); (3) optional LBVS or pharmacophore triage; (4) docking (HTVS/SP/XP) with consensus rescoring; (5) post-docking filtering (strain, protein–ligand contacts, synthetic tractability, novelty); and (6) selection for synthesis and experimental validation. Iterative cycles then integrate cheminformatics clustering, ADMET prediction, and medicinal chemistry feedback to converge on tractable chemotypes. For SBVS, widely used docking engines include Glide (HTVS/SP/XP), AutoDock Vina, DOCK, GOLD, and others; physics-based free-energy refinements (e.g., FEP+) increasingly sit downstream of docking for potency ranking among close analogs. LBVS and field-based methods (e.g., from Cresset) complement docking with shape/electrostatic similarity and pharmacophore queries; deep-learning toolkits and benchmarks (e.g., GuacaMol, MOSES) support rapid model development for activity prediction and generative design. Enterprise platforms (e.g., Schrödinger VS workflows) orchestrate these steps with parallelization. While cloud/HPC solutions enable elastic scaling when libraries exceed 10^8^ compounds. Campaigns docking 10^8^–10^9^+ molecules have uncovered entirely new chemotypes against diverse targets, demonstrating that scale itself is a discovery lever [64]. docked ~170 million make-on-demand compounds, experimentally confirming nanomolar hits with novel scaffolds at AmpC β-lactamase and D4 receptor. Open-source pipelines such as VirtualFlow subsequently industrialized billion-molecule SBVS on commodity cloud/HPC, providing turnkey workflows for pre-filtering, docking, and results management. Elastic compute has moved VS from fixed clusters to cloud-native scheduling and GPU acceleration, improving wall-clock throughput while enabling iterative enrichment strategies (e.g., quick-and-cheap docking→focused high-precision rescoring). This has normalized “campaigns” that iterate between computational enrichment and make-on-demand synthesis within weeks rather than months. Machine learning now prioritizes library regions before docking, reducing computation by several orders of magnitude. “Deep Docking” learns from an initial sparse docking pass to triage the study of an ultra-large library, while independent deep-learning studies have prospectively discovered active chemotypes (e.g., halicin as a novel antibiotic) from millions of candidates. In parallel, benchmark suites (GuacaMol, MOSES) have standardized evaluation of generative and predictive models used to guide VS.

Computational hits must be confirmed experimentally (biochemical/biophysical assays), then winnowed by developability. Orthogonal confirmation (e.g., thermal shift, SPR, ITC, crystallography/cryo-EM) helps verify binding mode and rule out assay artifacts. Prospective triage integrates in silico ADMET (solubility, permeability, metabolic stability) and liability filters. Classical guidelines (Rule-of-Five; polar surface area/rotatable bonds) remain useful first-pass heuristics, though modern practice couples them with model-based predictions and medicinal chemistry.

## 8. Future Directions and Role of AI in Drug Discovery

Multimethod pipelines Pharmacophore → Docking → MD → Free Energy yield better decision-quality than any single technique. Pharmacophore queries (ligand- or structure-based) rapidly focus libraries on interaction hypotheses. Docking then proposes concrete poses consistent with the pharmacophore and receptor geometry. Short MD relaxations stabilize induced-fit states, identify conserved waters, and flag unstable poses; consensus contact/energy metrics prune false positives. Finally, relative binding free energy (e.g., FEP+) resolves tight structure activity relationship within a chemotype, ranking analogs at the ~1 kcal·mol^−1^ level to drive synthesis. Structural coverage is the linchpin of SBVS, and breakthroughs in structure prediction now “unlock” targets formerly inaccessible to docking. AlphaFold and related methods deliver atomic-level models that, after limited refinement, can seed docking/MD workflows when experimental structures are unavailable. The introduction of AlphaFold 3 and diffusion-based modeling approaches represents a paradigm shift in computational drug discovery. Unlike earlier versions, AlphaFold 3 can model multi-component biological systems, including ligand binding and macromolecular interactions, thereby bridging the gap between static structure prediction and functional molecular recognition [72]. In practice, teams assess multiple AF2 models/ensembles, apply binding-site refinements (e.g., side-chain repacking, MD sampling), and validate emergent poses with pharmacophore consistency and experimental structure–activity relationship. Integration is not only sequential but adaptive. AlphaFold 3 and diffusion-based modeling approaches represent major advances toward modeling multi-component biological systems. However, challenges remain in achieving consistent accuracy for protein–ligand interactions, nucleic acid complexes, and post-translational modifications. Continued integration with molecular dynamics, docking, and experimental validation will be essential to fully realize their potential in drug discovery workflows. AI-guided enrichment loops interleave with physics-based steps: an initial docking subset trains a model (e.g., Deep Docking) that triages the remaining library; top predictions are re-docked, MD-filtered, and funneled to synthesis. At a larger scale, such iterations can be parallelized using VirtualFlow-style orchestrators across thousands of cores/GPUs, with chemoinformatics clustering for scaffold diversity. The consequence is higher hit rates and chemotypes per compound synthesized compared with commonly used fixed-screen strategies. In addition to conventional machine learning approaches, recent advances have led to the development of integrated artificial intelligence (AI) and large language model (LLM)-driven platforms for drug discovery [73,74]. Recent advances have led to the emergence of integrated AI- and large language model (LLM)-driven platforms for drug discovery, including systems such as PharmAgents, FROGENT, LIDDiA, AgentD, and AutoBinder Agent. The objectives of these platforms are to integrate various steps in the drug discovery process by integrating generative modeling, cheminformatics, molecular design, and automated reasoning in end-to-end or closed-loop applications. With the help of LLMs, they can aid in hypothesis generation, suggest new chemical structures, and refine candidates through iterative feedback and refinement of candidate properties and feedback loops, shortening the design-make-test cycle. Although promising, there are a number of challenges. It remains not easy to rationalize proposed molecules or mechanisms as the interpretation of model decisions remains limited. The quality and variety of training data are important to the reliability and generalizability of predictions, bringing up the issue of bias and robustness. Moreover, benchmarking systems of measuring these platforms are yet to be standardized, making it difficult to compare methods objectively. Above all, predictions provided by these systems must be hardened by experimental validation since in silico performance is not necessarily translated into biological activity. Thus, though the platforms developed with the help of LLM are an important step in the direction of autonomous drug discovery, they can be considered as supplementary tools that complement, but do not substitute, developed computational and experimental strategies [75,76,77]. These platforms operate within closed-loop frameworks that integrate computational prediction with iterative feedback, significantly improving efficiency, scalability, and hit identification in modern drug discovery pipelines.

Deveral studies demonstrate this synergistic effect: (i) the ultralarge efficacy data set of high-throughput SBVS, rapid one-stoichiometry synthesis of hits on demand, and iterative lead optimization to the nanomolar range, proving that scale plus incremental improvement yields first-in-class scaffolds. During medicinal chemistry cycles, FEP+ has prospectively influenced analog selection and reduced the synthesis workload by zooming in on potentially the most promising substitutions. Docking scores do not correlate well with affinity, and enrichment depends on the target/protein state. Consensus scoring, physics-based rescoring (MM/GBSA or FEP), and MD-derived in silico stability only partially abrogate this gap (Table 5). For example, VS results are highly dependent on the curation of protein structures, protonation/tautomer states, and assay descriptors. In response to community guidance, we prioritize careful scrubbing of chemical/biological data, open workflows, and notebooks/containers that can be re-run for reproducibility and reuse.

**Figure 5:**
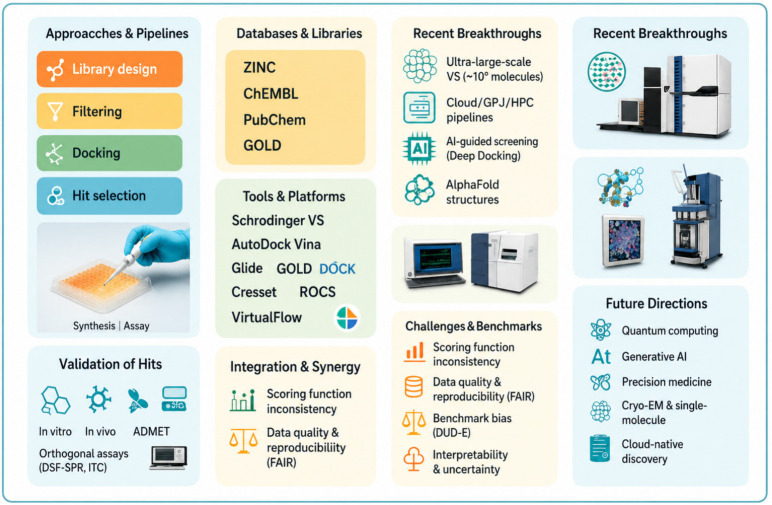
Approaches, libraries, tools, breakthroughs, validation, integration, challenges, and future directions. Boxes, arrows, and photographs summarize workflows from library design and docking to assay confirmation, considerations enabling discovery.

The FAIR data principles also advocate for findable, accessible, interoperable, and reusable datasets and models. Popular enrichment benchmarks such as DUD-E catalyzed method development but also introduced analog/decoy biases that can inflate performance estimates; careful benchmark selection and “bias-controlled” tests are essential. When possible, prospective validation or blinded external sets should be used to verify generalization. As AI prioritizes larger fractions of libraries, interpretability (e.g., pharmacophore/interaction attributions), uncertainty quantification, and documentation of training data become central for decision-making, auditing, and regulatory dialogue. VS increasingly draws on integrated public/private data; teams should respect licenses, privacy constraints, and data-sharing policies while adhering to FAIR/traceability practices. Quantum algorithms promise advantages for electronic-structure problems central to binding and reactivity, potentially improving the fidelity of scoring and induced-fit modeling. Near-term devices (via VQE/ADAPT-VQE) and error-mitigated simulations target small active sites and fragments; for the medium term, hybrid quantum-classical workflows may refine docking poses or parameterize bespoke interactions that challenge classical force fields. Although timelines remain uncertain, the trajectory suggests domain-specific quantum acceleration will complement rather than replace classical VS/MD. Foundation models and reinforcement learning increasingly propose synthetically accessible, property-constrained molecules that meet multi-parameter objectives before docking/MD. Community benchmarks (GuacaMol, MOSES) have improved rigor in evaluating distribution learning, validity, novelty, and goal-directed optimization (Table 6). The emerging best practice is closed-loop design: generate→triage by QSAR/ADMET→dock/score→MD refine→pick for synthesis→feedback assay data to retrain. Integration of patient-derived omics, perturbation signatures (e.g., the next-generation Connectivity Map), and target structures will enable individualized VS campaigns: predict vulnerabilities from transcriptomic/proteomic profiles, prioritize compounds with matched mechanisms, and validate in patient-derived models (Figure 5). Computational scaling and causal modeling will be key to translating this promise to the clinic. Routine near-atomic cryo-EM now resolves dynamic states and ligandable cryptic pockets for previously intractable targets. Combining cryo-EM ensembles with MD-derived conformational sampling supports ensemble docking and pocket-state-aware VS, especially for allosteric and membrane proteins. The future of VS is elastic: orchestrators that spin up tens of thousands of CPU/GPU cores on demand, autoscale storage and databases, and couple active learning with make-on-demand synthesis vendors. Platforms such as VirtualFlow already demonstrate billion-scale SBVS with modular pre-/post-filters, while integration with enterprise cheminformatics ensures traceable data lineage from screen to clinic.

## 9. Conclusions

Recent advances across structure-based and ligand-based approaches have reshaped small-molecule discoveries. Docking engines sampled poses more efficiently and, when coupled with consensus or physics-grounded rescoring and explicit treatment of waters, delivered stronger early enrichment. Pharmacophore modeling had progressed from geometric templates to energy-weighted and ensemble hypotheses that captured receptor plasticity and enabled credible scaffold hopping. Molecular dynamics routinely reached microsecond scales on accessible hardware, revealed cryptic pockets, quantified water networks, and supported endpoint and alchemical free-energy calculations suitable for prospective decisions. Ultra-large virtual screening became practical through curated make-on-demand libraries, elastic computing, and reliable workflow automation, allowing campaigns across hundreds of millions of candidates. High-accuracy structure prediction broadened the tractable target set and, after limited refinement, supported routine structure-based campaigns. The greatest gains arose when methods were combined rather than used alone. Libraries shaped by medicinal-chemistry rules and pharmacophore filters were docked at scale, then triaged by contact patterns, strain energy, synthetic tractability, and diversity. Short dynamics run stabilized promising poses, discarded strained ones, and supplied trajectory-derived pharmacophores. For closely related analogs, rigorous free-energy calculations ranked substitutions at decision-making resolution and reduced avoidable synthesis. Throughout, biophysical confirmation, structural follow-up, and cellular assays closed the loop from computation to experiment. This integrated cycle had shortened design–make–test timelines, increased hit novelty, and improved the credibility of advancement decisions. Sustained progress depended on standardization, transparency, and collaboration. Preparation protocols, protonation and tautomer rules, and threshold criteria were documented, versioned, and shared. Benchmarks addressed bias and emphasized prospective validation with metrics for early recognition and scaffold novelty. Datasets, models, and code followed FAIR principles to enable reuse and independent checks. Finally, partnerships across academia, industry, and computing providers aligned with open libraries, make-on-demand synthesis, and orthogonal assay panels have turned scalable computation into reproducible medicines with greater efficiency and lower cost.

## Figures and Tables

**Table 1:** Core computational approaches and where they fit in drug discovery programs.

Modality	Primary Goal	Typical Inputs	Key Outputs	Strengths	Main Limitations	Representative Tools	Citation(s)
Molecular docking	Predict binding mode and rank candidates	Prepared protein (X-ray/cryo-EM/AF2), curated ligands, microstates	Poses, interaction maps, docking scores	Fast triage; structure-aware hypotheses	Scoring/solvation approximations; limited receptor flexibility	AutoDock Vina, Glide, GOLD, DOCK	[22,29,30]
Pharmacophore modeling	Capture essential 3D features for activity	Active ligands and/or protein–ligand complexes	Feature hypotheses; 3D queries	Scaffold hopping; ultra-fast pre-filtering	Overfitting risk; conformer/feature quality sensitive	PHASE, LigandScout, MOE, PharmaGist	[27,31,32]
Molecular dynamics (MD)	Probe flexibility, water networks, pose stability	Protein–ligand complex, force field, solvent/ions	Trajectories, RMSD/RMSF, H-bond/water analyses; MM-GB/SA/FEP	Mechanistic insight; validates poses; supports ΔΔG	Sampling cost; FF/setup sensitivity	CHARMM36m, AMBER ff14SB, OPLS3e	[23,33,34,35]
Virtual screening (VS)	Prioritize hits from ultra-large libraries	ZINC/ChEMBL/PubChem/GDB; docking/LBVS filters	Ranked shortlists; clustered chemotypes	Scales to 10^8^–10^9^; cloud/HPC ready	Benchmark bias; hit confirmation required	VirtualFlow; LBVS + SBVS pipelines	[36,37,38,39]

**Table 2:** Software, platforms, and data resources used across workflows.

Category	Tool/Resource	License	Notable Capabilities	Typical Scale	Citation
Docking engine	AutoDock Vina	Open source	Stochastic search; empirical scoring; multithreaded	10^5^–10^7^	[52]
Docking engine	Glide (HTVS/SP/XP)	Commercial	Hierarchical filters; pose refinement; XP scoring	10^5^–10^7^	[22]
Docking engine	GOLD	Commercial	GA search; side-chain flexibility; constraints	10^5^–10^6^	[53]
Docking engine	DOCK	Open source	Shape/grid; anchor-and-grow; modular	10^5^–10^7^	[29]
Protein–protein docking	RosettaDock (server)	Academic	Rigid-body + side-chain optimization; web access	Interface sets	[42]
Pharmacophore	PHASE	Commercial	Common-pharmacophore ID; 3D-QSAR	10^6^–10^8^ (filter)	[27]
Pharmacophore	LigandScout	Commercial/academic	Feature extraction from complexes; 3D queries	10^6^–10^8^	[54]
Suite	MOE (+ LowModeMD)	Commercial	Conformers; pocket/feature tools; induced-fit aids	Project-scale	[31]
Orchestrator (VS)	VirtualFlow	Open source	Billion-scale docking; engine-agnostic; cloud/HPC	10^8^–10^9^+	[39]
Library	ZINC20/22	Free	Purchasable make-on-demand; ready-to-dock	10^9^-class	[36]
Library	ChEMBL	Free	Assay-annotated activities; targets	Millions of activities	[40]
Library	PubChem	Free	Open compounds and bioassays	100M+ compounds	[55]
Enumerated universe	GDB-17	Free	166B enumerated molecules	10^11^+	[56]
Structures	Protein Data Bank (PDB)	Free	Standardized macromolecular structures	200k+ entries	[24,25]
Benchmark	DUD-E	Free	Actives/decoys for many targets	Benchmark sets	[37]
Benchmark critique	DUD-E bias analysis	Free	Analog/decoy bias diagnostics	Benchmark audit	[57]
Structure prediction	AlphaFold	Free for models	Near-experimental single-chain models	Proteome-scale	[41]
ML benchmarks	GuacaMol/MOSES	Open source	Generative design benchmarking	Model-dependent	[58,59]

**Table 3:** Drugs delivered with computational pipelines (docking/pharmacophore/MD/VS).

Drug (Year)	Target/Indication	Primary Computational Technique(s) Credited	What the Pipeline Contributed (Very Brief)
Captopril (1981)	ACE/hypertension	Early structure-based design guided by carboxypeptidase-A models	Transition-state/active-site modeling yielded thiol-bearing inhibitors with oral activity
Zanamivir (1999)	Influenza neuraminidase/influenza	SBDD + docking on NA crystal structures	Rational modifications to sialic-acid scaffold to exploit NA catalytic site; first-in-class NA inhibitor
Saquinavir (1995)	HIV-1 protease/HIV	SBDD from protease–peptide complexes	Transition-state mimic designed from active-site geometry; launched first HIV PI
Indinavir (1996)	HIV-1 protease/HIV	SBDD with iterative docking/optimization	Optimized P1/P2 groups for S1/S2 subsites; improved oral PK
Ritonavir (1996)	HIV-1 protease/HIV	SBDD (crystallography-guided)	Potent PI that became PK booster after metabolic insights
Dorzolamide (1995)	Carbonic anhydrase II/glaucoma	SBDD	Active-site geometry (Zn^2+^ coordination) guided sulfonamide design
Tirofiban (1998)	Integrin αIIbβ3/ACS	SBDD/mimetic design from RGD-ligand structures	Crystal structures with tirofiban/eptifibatide informed small-molecule antagonist design
Aliskiren (2007)	Renin/hypertension	SBDD + modeling on renin structures	Non-peptidic scaffold engineered for S1/S3/S1′ pockets; oral renin inhibitor
Boceprevir (2011)	HCV NS3 protease/HCV	SBDD	Warhead and P1/P2 optimization for covalent reversible inhibition
Rivaroxaban (2011)	Factor Xa/anticoagulation	SBDD + crystallography	Structure-guided optimization and FXa co-crystal analysis supported binding-mode tuning
Baloxavir marboxil (2018)	Influenza cap-dependent endonuclease/influenza	SBDD on PA endonuclease	Metal-chelation pharmacophore and pocket mapping drove first-in-class CEN inhibitor

**Table 4:** Scoring families, force fields, and free-energy methods (what they model and when to use).

Domain	Method/Model	What It Modeled/ Optimized	Strengths	Limitations	Typical Use	Citation
Docking scoring (empirical)	GlideScore; AutoDock4/Vina	vdW, H-bonding, desolvation terms fit to data	Fast; good enrichment	Transferability limits	Primary SBVS ranking	[22]
Docking scoring (knowledge-based)	Statistical PMF/X-Score (generic)	Statistical atom–atom potentials	Simple; robust	Coarse physics	Complementary rescoring	[57,65,66]
Docking scoring (ML)	RF-Score; NNScore; GNINA CNN	Data-learned pose/affinity from structures	Captures nonlinearity; strong top-N	Needs curated data; bias risk	Rescoring top poses	[44]
Physics-like ranking	MM-PB/GBSA	Continuum solvation + force field	Interpretable; fast triage	Dielectric/entropy sensitive	Post-docking triage	[61]
Alchemical ΔΔG	FEP (RBFE)	Relative binding free energy	~1 kcal·mol^−1^ resolution	Setup/sampling cost	Lead optimization	[67]
Alchemical ΔG analysis	Thermodynamic integration	Gradient-based alchemy (λ windows)	Rigorous; general	Complex setup	Tight Structure activity relationship decisions	[68]
Force field (proteins)	CHARMM36m	Folded and IDP proteins	Balanced backbone/IDP	χ issues for some residues	General MD	[33]
Force field (proteins)	AMBER ff14SB	Proteins	Updated side-chain/backbone	Needs matched ligand params	General MD	[34]
Force field (proteins/ligands)	OPLS3e	Drug-like ligands + proteins	Broad ligand coverage	Licensed	Lead optimization MD	[35]
Water models	TIP3P; TIP4P-Ew	Solvent representation	Standardized hydration	Model-specific limits	Routine MD	[69,70,71]

**Table 5:** Future directions and role of AI in drug discovery.

Direction	What AI/Tech Adds	Concrete Example(s)	Expected Impact
Ultra-large virtual screening (10^8^–10^9^+ molecules)	Cloud/HPC orchestration; adaptive scheduling	VirtualFlow enables billion-scale SBVS, modular docking stacks	Orders-of-magnitude expansion of search space; more novel chemotypes
AI-guided triage for VS	Learn from sparse docking to skip most of the library	Deep Docking cuts compute by ~50×; consensus/pose filters downstream	Same hit rate at fraction of cost/time
DL-rescoring and pose selection	CNN/GDL models refine ranks/poses post-docking	GNINA family improves top-n pose accuracy	Better early enrichment; fewer false positives
Structural coverage via AI	High-accuracy protein structures when experiments lack	AlphaFold proteome-scale structures	“Unlocks” SBDD for previously intractable targets
Bias-aware benchmarking	Detect spurious dataset signals in training/validation	DUD-E bias analysis cautions DL claims	More reliable, reproducible VS metrics
Omics-driven personalization	Match compounds to patient/pathway signatures	CMap/LINCS L1000 profiles (1.3M signatures)	Indication selection, MoA inference, repurposing
Cryo-EM + MD + ensemble docking	Multi-state targets and cryptic pockets	State-aware docking/MD on EM ensembles	Allosteric/drugging “undruggables”
Foundation models and generative design	Rapid de novo ideas under multi-param constraints	GuacaMol/MOSES benchmarks standardize eval	Tighter design-make-test loops

**Table 6:** Validation metrics, assays, library filters, and practical mitigations.

Stage	Item/Metric/Assay	Measures/Goal	Practical Notes/Action	When to Use	Citation
Benchmarking	EF1%, EF5%, BEDROC, ROC-AUC	Early recognition and global ranking	Emphasize EF/BEDROC for top-fraction testing	Retrospective method evaluation	[37]
Benchmark audit	DUD-E bias checks	Analog/decoy bias detection	Use bias-controlled splits; external sets	Before claiming generalization	[57]
Orthogonal confirmation	SPR	kon, koff, KD	Control surface artifacts; kinetics insight	Hit/lead confirmation	[78]
Orthogonal confirmation	ITC	ΔH, ΔS, KD, stoichiometry	Thermodynamics; gold standard	Characterize prioritized hits	[79]
Orthogonal confirmation	MST	KD in solution	Low sample; buffer-flexible	Cross-validate binding	[80]
Mode validation	X-ray/cryo-EM	Bound structure and binding mode	Deposit to PDB for reuse	Structural follow-up	[81]
Library filter	Rule-of-Five	Oral developability	Use as soft gate with medicinal review	Pre-screening	[82,83]
Library filter	Veber criteria	Permeability/bioavailability	PSA/rotor thresholds	Pre-screening	[84,85]
Library filter	PAINS	Remove assay-interference chemotypes	Avoid over-filtering true actives	Library assembly	[86]
Library filter	Brenk alerts	Remove problematic fragments	Combining with expert review	Library assembly	[87]
Data stewardship	FAIR principles	Reproducibility and reuse	Version inputs; share code/models	All stages	[88]
Curation	Chemogenomics checklists	Correct labels/states/microstates	Scripted, versioned prep	Before modeling/VS	[89]
Common pitfall	Mis-protonated residues/ligands	Spurious contacts/energies	Enumerate microstates; pKa review	Prep and docking/MD	[89]
Common pitfall	Ignored conserved waters	Missed bridges/affinity	Retain/map waters; test displacement	Docking triage	[23]
Common pitfall	Metal coordination errors	Unrealistic poses/instability	Add constraints; spot-check with QM/MM	Targets with metals	[61]
Common pitfall	Overfitting benchmarks	Inflated enrichment	Prospective tests; external sets	Method claims	[90,91,92]

## Data Availability

No new data were created or analyzed in this study. Data sharing is not applicable.
